# Sister Mary Joseph nodule as cutaneous manifestations of metastatic ovarian cancer: A case report and review of the literature

**DOI:** 10.1097/MD.0000000000028712

**Published:** 2022-02-11

**Authors:** Xianglin Nie, Xing Chen, Yi Jiang, Yi Zhong, Ting Chen, Wenjun Cheng

**Affiliations:** a Department of Gynecology, The First Affiliated Hospital with Nanjing Medical University, Nanjing, China; b Department of Radiology, The First Affiliated Hospital with Nanjing Medical University, Nanjing, China.

**Keywords:** ovarian cancer, Sister Mary Joseph's nodule, umbilical mass

## Abstract

**Rationale:**

The Sister Mary Joseph's nodule is an umbilical nodule resulting from the metastasis of malignant tumors in the pelvic and/or abdominal cavity. Sister Mary Joseph's nodules are very rare, and the morphology of the skin lesions is not specific and is easily misdiagnosed. Here, we report a case of cutaneous manifestations of metastatic ovarian cancer.

**Patient concerns:**

The patient was admitted to our hospital because of abdominal distention, and a nodule was found in the umbilicus. A computerized tomography scan of the entire abdomen showed nodular soft tissue in the subcutaneous fat space of the umbilical area and multiple pelvic masses, which were suspected metastases of peritoneal and omentum ovarian cancer.

**Interventions:**

To confirm the pathological diagnosis, posterior fornix puncture was performed. Pathological biopsy showed adenocarcinoma. Histological examination revealed a mass arising from high-grade serous carcinoma of the ovary. The patient received 2 cycles of chemotherapy with paclitaxel liposomes and carboplatin and underwent interval debulking surgery. Postoperative pathology was consistent with high-grade serous carcinoma of the ovary. Cancer involvement was observed in umbilical lesions. After the operation, the patient was given 6 cycles of chemotherapy with paclitaxel liposomes and carboplatin.

**Outcomes:**

The patient underwent follow-up until October 2020. A computerized tomography scan of the entire abdomen showed that the lymph nodes in the abdominal cavity were larger than before, suggesting a platinum-sensitive relapse. After receiving the same regimen of chemotherapy, carbohydrate antigen 125 dropped to the normal range, and consolidated treatment was administered for 3 cycles. Owing to her BRCA1 mutations, olaparib was administered for maintenance treatment. Until now, she had been in the outpatient clinic for regular follow-up visits.

**Lessons:**

The umbilicus remains an infrequently examined area, which cannot be underestimated and warrants careful clinical follow-up and histological evaluation, as appropriate.

## 1 Introduction

Sister Mary Joseph's nodule (SMJN) is an umbilical lesion caused by intra-abdominal and/or pelvic tumor metastasis.^[1]^ It was named after Sister Mary Joseph, a surgical assistant at the Mayo Clinic, who pointed out to the surgeon that umbilical nodules may be one of the signs of metastasis of malignant abdominal and pelvic tumors. Common umbilical lesions can be classified as benign and malignant lesions, including benign umbilical hernia, congenital lesions, umbilical granuloma, umbilical polyps, umbilical abscess, umbilical cyst, etc.^[2,3]^ Umbilical lesions need to be carefully examined because such lesions may reflect serious hidden diseases or malignant tumors. Previous reports have shown that 57% of umbilical nodules are benign. Nevertheless, almost all malignant lesions were secondary tumors.^[4]^ About 25% of the cases are related to gynecological malignancies, mainly ovarian cancer, which has the highest case fatality rate among malignant tumors of the female reproductive system.^[5]^ SMJN is a significant symptom on physical examination. Sometimes, it is the only clinical sign of an intra-abdominal and/or pelvic tumor.

Herein, we report a case of SMJN as a cutaneous manifestation of metastatic ovarian cancer. Owing to the special clinical clue and its clinical significance, it deserves our attention. The patient provided informed consent, and patient anonymity was preserved.

## 2 Case presentation

**Figure 1:**
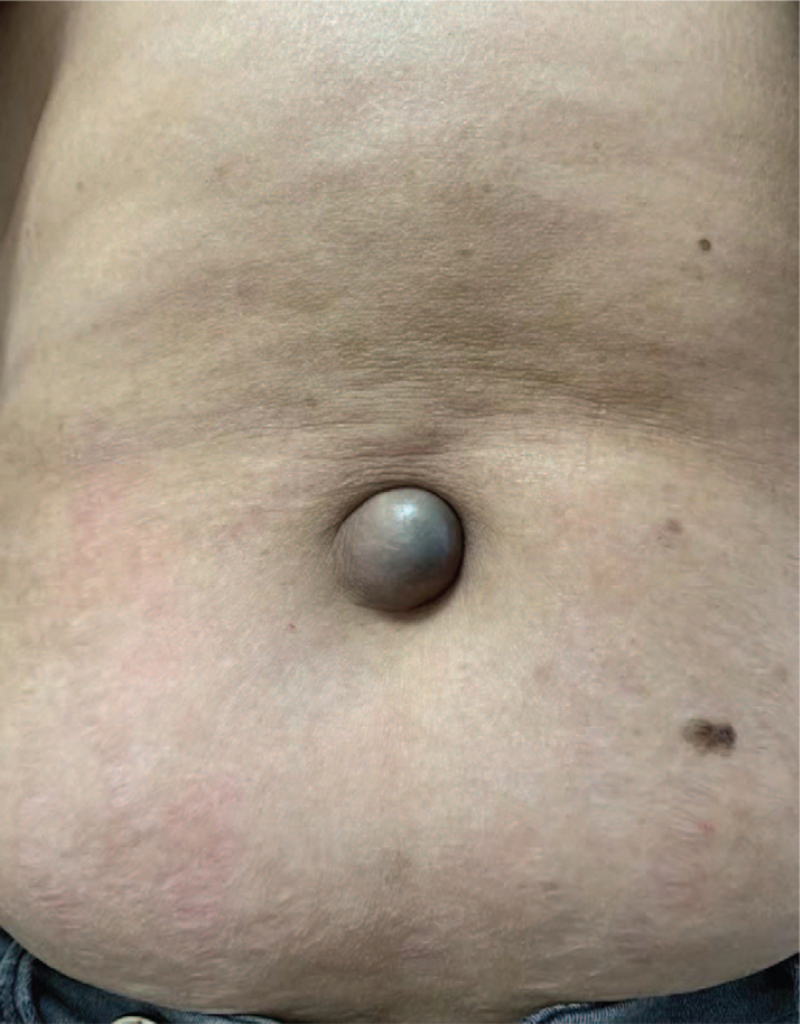
The nodule in the umbilicus.

**Figure 2:**
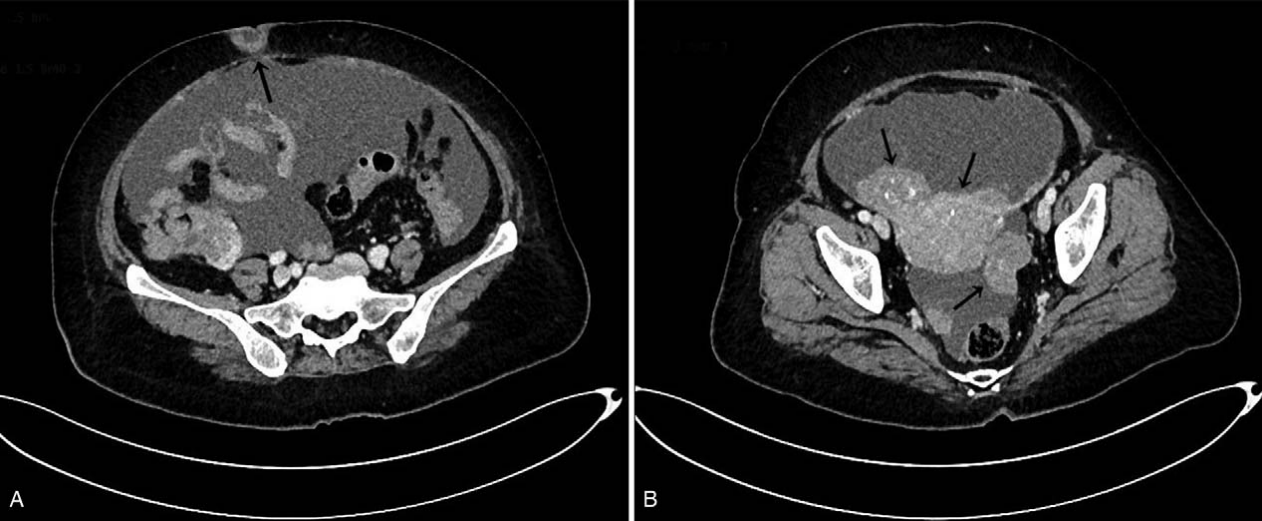
Computerized tomography (CT) scan of the entire abdomen. A nodular soft tissue in the subcutaneous fat space of the umbilical area was observed (A). Furthermore, multiple pelvic masses which were suspected metastases of peritoneal and omentum of ovarian cancer were shown (B).

The patient was 62-year-old, who has experienced natural menopause for 10 years. In September 2017, a blood test revealed a low level of carbohydrate antigen 125 (CA125) (80 U/ml). Eight months later, she was admitted to our hospital because of abdominal distention, and a nodule was found in the umbilicus (Fig. 1). She rechecked CA125, which was up to 1252U/ml. A computerized tomography (CT) scan of the entire abdomen in May 2018 showed nodular soft tissue in the subcutaneous fat space of the umbilical area (Fig. 2A) and multiple pelvic masses, which were suspected metastases of peritoneal and omentum ovarian cancer (Fig. 2B). To confirm the pathological diagnosis, posterior fornix puncture was performed. Pathological biopsy showed adenocarcinoma. Abnormalities on gastrointestinal endoscopy were not found. Considering the widespread dissemination of the tumor, a primary debulking surgery could not be performed. The patient received 2 cycles of chemotherapy with paclitaxel liposomes and carboplatin. After 2 cycles of chemotherapy, re-examination of CT showed that the lesion was smaller than before, but the density of the soft tissue in the umbilical area was roughly the same. In August 2018, the patient underwent interval-debulking surgery. Postoperative pathology was consistent with high-grade serous carcinoma of the ovary. Cancer involvement was observed in umbilical lesions. After the operation, the patient was given 6 cycles of chemotherapy with paclitaxel liposomes and carboplatin. The CA125 dropped to 49.6 U/ml after the operation, and it dropped to normal range (< 35 U/ml) after the first cycle of chemotherapy. The patient was followed up until October 2020. Serological examination revealed that CA125 level of 51 U/ml. A CT scan of the entire abdomen showed that the lymph nodes in the abdominal cavity were larger than before, suggesting recurrence. Combined with the patient's medical history, a platinum-sensitive relapse was observed. After receiving the same regimen of chemotherapy, CA125 dropped to the normal range, and consolidated treatment was administered for 3 cycles. Owing to her BRCA1 mutations, olaparib was administered for maintenance treatment. Until now, she had been in the outpatient clinic for regular follow-up visits. There were no abnormalities in the ultrasound or tumor indicators.

## 3 Discussion

Ovarian cancer is the most lethal gynecological malignancy. It was estimated that the number of new cases of ovarian cancer in 2020 was as high as 21,750, and the estimated number of deaths was as high as 13,940.^[6]^ It is difficult to detect because the ovary is located in the deep pelvic cavity, and the patients have no early symptoms. It has been reported that over 70% of patients have metastasis outside the ovary at the time of diagnosis. They usually spread directly into the peritoneal cavity. Distant metastases may also occur at the time of ovarian cancer diagnosis (stage IV disease) or arise during disease evolution. The most common sites are the pleura, liver, lungs, and lymph nodes (LNs).^[7]^ Skin metastases are rare, occurring in 0.9% to 4% of patients.^[8]^ Skin metastases are classified as metastatic umbilical tumors, which are known as SMJNs, or non-SMJN skin metastases. The case reported above was about the SMJN.

SMJN is a rare clinical finding that accounts for up to 3% of all abdominal and pelvic malignancies. A typical SMJN is a firm, irregular umbilical nodule, averaging between 1 cm and 1.5 cm in size, and sometimes up to 10 cm in diameter.^[9]^ The nodules are usually described as white, bluish purple, or red, which are sometimes painful and pruritic. The surface may be cracked, ulcerated, or necrotic, may have bloody, mucinous, serous, or purulent secretions, and may even be normal.^[9]^ It is frequently overlooked or misinterpreted during physical examinations. Galvan reviewed 407 umbilical tumor cases, the most common of which were in the stomach (23%), ovary (17%), colon and rectum (15%), pancreas (9%), and uterus (6%). In women, ovarian cancer is the most common origin of SMJN; 42% to 47.7% of women with SMJN have ovarian cancer.^[10]^

Ultrasound can be applied to detect umbilical nodules and monitor the tumor recurrence.^[11]^ When umbilical nodules are found, it is necessary to make an accurate histological diagnosis of primary and metastatic lesions. Fine needle aspiration biopsy is the gold standard, which is safe, rapid, and inexpensive. However, umbilical nodule puncture was not performed. Gynecological examination revealed nodules in the posterior fornix of the vagina, and a hard mass was palpable at the appendage. Combined with high levels of CA125 and ascites, ovarian cancer can be highly suspected. Of course, the pathology of the posterior fornix puncture also suggests serous carcinoma of ovarian origin. Imaging examinations after 2 courses of chemotherapy showed that the umbilical nodules were not smaller than before, but the abdominal and pelvic lesions were smaller than before. It can be seen that metastatic umbilical nodules are not very sensitive to chemotherapy, and surgical resection seems to be more effective.

The mechanism of umbilical seeding from primary tumors remains unclear. However, several anatomical standards and hypotheses have been proposed. The contiguous spread from the intraperitoneal metastasis to the umbilicus appears to be the most common mechanism of SMJN occurrence, where there is a relatively weak depression in the anterior abdominal wall, located at the level of the highest point of the iliac crest, opposite to the L3-L4 vertebral body or L4 vertebral bodies.^[3]^ The formed tumor cells fall off into the peritoneal cavity, where they float in the peritoneal fluid and are carried throughout the peritoneal cavity, attach to adjacent peritoneal organs, and eventually form metastatic tumors. Relying on gravity, large volumes of tumor accumulate within the umbilical cul-de-sac.^[12]^ In addition, SMJNs may also spread via lymphatic channels to the pelvic and para-aortic nodes, and less to the inguinal or supraclavicular nodes. Four sets of lymphatics pass from the umbilicus: axillary lymph nodes, external inguinal nodes, along the falciform ligament, and deep inguinal nodes. Therefore, another theoretical metastatic pathway involves retrograde lymph flow along the inguinal lymph nodes or lymph nodes of the falciform ligament. Hematogenous metastasis is another type of metastasis. Nevertheless, in the absence of a large number of other blood-borne metastases, hematogenous spread to this site is highly unlikely, and SMJNs via the hematogenous pathway are very rare.^[13]^ There are other routes to the umbilicus as well. For example, the obliterated umbilical arteries and urachus could also provide pathways; however, there is almost no literature associated with urinary tract malignancy with umbilical involvement.^[14]^

## 4 Conclusion

In conclusion, significant progress has been made in elucidating the role of BRCA1 and BRCA2 and their mutations in the risk and prognosis of epithelial ovarian cancer. However, there are no detailed studies on the location of recurrence or mechanism of metastasis in patients with BRCA-mutated ovarian cancer. Here, we describe a case of OC with BRCA1 mutation and umbilical metastases. Therefore, this finding may have crucial implications for the metastatic pattern and clinical management of BRCA-mutant OC.

## Author contributions

XN and XC wrote the manuscript. YJ, TC, and YZ contributed to data collection and analysis. WC contributed to data interpretation and critical revision. All the authors have read and approved the manuscript.

**Conceptualization:** Xianglin Nie, Xing Chen.

**Data curation:** Yi Jiang, Ting Chen, Yi Zhong.

**Formal analysis:** Yi Jiang, Ting Chen.

**Supervision:** Xing Chen, Wenjun Cheng.

**Writing – original draft:** Xianglin Nie.

**Writing – review & editing:** Xing Chen, Wenjun Cheng.
